# A narrative review of interventions for post-stroke frailty: current advances and future directions

**DOI:** 10.3389/fneur.2025.1592797

**Published:** 2025-07-08

**Authors:** Xiaowen Fan, Yi Xia, Shengwang Xu, Shulei Jia

**Affiliations:** ^1^School of Nursing, Nanchang University, Jiangxi Medical College, Nanchang, Jiangxi, China; ^2^Department of Breast Surgery, The Second Affiliated Hospital of Nanchang University, Nanchang, Jiangxi, China

**Keywords:** frail, stroke, intervention, review, exercise, nutrition

## Abstract

Frailty affects the health outcomes of stroke patients. Timely identification of the progression of frailty in stroke patients and intervention play a crucial role in prognosis. This article reviews domestic and international literature on frailty interventions for stroke patients, focusing on the definition of stroke combined with frailty, frailty staging, and the effects of exercise, nutritional, cognitive, and psychosocial interventions, and medication management on the prognosis of stroke. The aim is to provide a reference for improving the prognosis of stroke patients with frailty.

## 1 Introduction

Stroke, commonly known as *cerebrovascular accident*, refers to a group of brain diseases caused by circulatory disorders leading to sudden localized or diffuse neurological deficits (1). Stroke has become the second leading cause of mortality in rural residents and the third in urban residents in China. In China, there are over 2 million new stroke cases annually, with the highest disability-adjusted life years (DALYs) among all diseases (2). Research indicates that stroke accelerates the progression of frailty, which in turn significantly impacts health outcomes in stroke patients. Frailty is linked to stroke severity, cognitive function, morbidity and mortality (3).

Frailty is defined as a nonspecific pathological state in older adults, characterized by a decline in physiological functional reserve, elevates bodily vulnerability and diminishes stress resistance. Which can lead to multisystem pathophysiological alterations (4). Moreover, frailty is a subnormal state in older adults between health and disease, which is not only a common clinical state after stroke, but also increases the risk of adverse health events such as falls, incapacitation, cognitive impairment and disability. Frailty is an important determinant of poor recovery after stroke (5).

Stroke patients presenting with symptoms such as reduced body mass, reduced grip strength, and slowed gait and meeting the diagnostic criteria for frailty is stroke combined with frailty (3), and it often leads to the occurrence of a range of adverse health outcomes such as prolonged hospitalization and reduced physical functioning (5, 6). According to Fried’s frailty phenotype, older adults can be classified into three frailty stages: robust, pre-frailty, and frailty (4). Among stroke patients, the prevalence of pre-frailty and frailty is 49 and 22%, respectively (7). For stroke patients with frailty, active interventions and long-term follow-up are critical to improving recovery outcomes, alleviating healthcare burdens, and reducing mortality. In recent years, the number of articles about frailty at home and abroad has been fluctuating, and exercise, nutrition, cognitive and psychosocial interventions for patients with frailty have become the hotspots of many scholars’ attention, and certain research results have been achieved. This article aims to synthesize existing research on the impact of interventions targeting frail patients with stroke, drawing from both domestic and international studies, with the objective of providing valuable insights to improve clinical outcomes and prognosis for stroke patients complicated by frailty.

## 2 Methods and analysis

In order to understand the progress of research on frail interventions for stroke patients, we conducted a review, synthesized and systematically analyzed relevant domestic and international papers.

The steps of the review are as follows.

### 2.1 Identifying relevant studies

This study examined articles published between January 2010 and December 2024, focusing on academic papers and theses on frailty interventions for stroke patients.

Search.

Search expressions were created based on the MeSH database with search terms including MeSH and free text.

(“Stroke”[Mesh] OR “Cerebrovascular Disorders”[Mesh] OR stroke[tiab] OR “cerebrovascular accident”[tiab] OR CVA[tiab]) AND (“Frailty”[Mesh] OR frail*[tiab] OR “physical frailty”[tiab] OR “geriatric syndrome”[tiab]) AND (interven*[tiab] OR therap*[tiab] OR management[tiab] OR treatment[tiab] OR rehabilitat*[tiab] OR “exercise therapy”[Mesh] OR “drug therapy”[Mesh]) AND (“2010/1/1”[Date - Publication]: “2024/12/31”[Date - Publication])

### 2.2 Study selection

There was no limit to the research design of the search literature, including Chinese and English search terms. Our systematic search protocol targeted publications from January 2010 to December 2024, executed during December 2024 across five multidisciplinary databases as follows: Web of Science, PubMed, CNKI, CQVIP, and Wanfang Data.

### 2.3 Type of intervention

#### 2.3.1 Exercise intervention

According to the *International Clinical Practice Guidelines: Identification and Management of Physical Frailty* developed by the International Conference on Frailty and Sarcopenia Research (ICFSR), physical activity interventions are recognized as the most effective first-line approach for preventing the onset of frailty and delaying its progression, receiving a “strong recommendation” rating (4). Additionally, exercise training is acknowledged as the primary method for frailty management (8). And it has been further demonstrated that the maximal effect of frailty improvement is obtained when frail older adults exercise 2–3 times per week for 50–60 min, 150–200 min per week, at an intensity of 3–4 (i.e., moderate ^1^) on the Borg CR-10 scale, and for at least 3–8 weeks (9).

Regarding current research on exercise interventions targeting frailty, studies can be categorized into the following types based on different criteria:

1. By intervention content: Single-component: Isolated modalities (e.g., resistance or aerobic training alone). Multi-component: Combined protocols (e.g., resistance + aerobic training, aerobic + balance training).
2. By exercise modality: Aerobic exercise, resistance training, balance training, flexibility exercises.
3. By delivery format: Tai Chi, Baduanjin, exergaming, elastic band exercises, walking, cycling, and multi-modal combined programs.
4. By exercise intensity: Low-, moderate-, and high-intensity regimens.
5. By intervention duration: Short-term (≤3 months) vs. long-term (>6 months) interventions.

Resistance training constitutes a cornerstone in frailty management by directly addressing sarcopenia—the primary pathophysiological mechanism underlying frailty. Furthermore, meta-analytic evidence demonstrates that structured resistance exercise not only enhances key physical parameters (muscle strength, gait speed, flexibility, postural stability, and physical performance) but also reduces depressive symptoms and frailty indices, collectively contributing to improved prognostic trajectories (10). Study confirms that resistance exercise intervention for frailty can improve the muscle strength of stroke patients, improve their neurological function, increase their bone density and balance, prevent falls, and delay muscle aging (11), which can effectively improve their quality of life and prolong the life cycle.

Aerobic exercise, also termed endurance training, demonstrates synergistic effects when integrated with other modalities to form multicomponent interventions. Notably, its combination with cognitive training enhances post-stroke neuroplasticity (12). As demonstrated by Li et al. (13), MotoMed cycling aerobic training combined with standard rehabilitation therapy enhances cardiovascular adaptation, neurological recovery, and activities of daily living (ADL) performance in elderly patients with acute ischemic stroke.

Balance and flexibility exercises include yoga and Traditional Chinese Exercises (TCEs) such as taijiquan, wuqiuquan, and baduanjin. Several studies have shown that tai chi improves balance function, mobility, step speed, functional stretching, and lower limb muscle strength in frailty older adults, and improves the quality of life of frailty older adults. A foreign meta-analysis (14) showed that evidence up to this point suggests that TCEs positively affects limb motor function, balance function, ADL ability, and neurological damage in stroke patients.

Apart from TCEs, the Otago Exercise Program (OEP), initially developed in the 1990s at New Zealand’s Otago Hospital under Campbell’s leadership (15) to mitigate fall risks in older adults, demonstrates efficacy in improving clinical outcomes for stroke survivors, particularly in fall prevention. This multicomponent intervention integrates warm-up routines, resistance and balance training, and aerobic walking. Empirical evidence (16) indicates that OEP enhances static and dynamic balance in this population through targeted lower limb strengthening and balance exercises, thereby ameliorating frailty severity and elevating both quality-of-life metrics and long-term prognosis.

In summary, multicomponent exercise interventions—particularly resistance-based combined modalities—demonstrate significant prognostic benefits for stroke survivors with frailty, aligning with evidence-based consensus and clinical practice guidelines (17, 18). As the primary approach for frailty management, resistance training not only enhances muscle strength and neurological recovery in this population but also increases bone density and optimizes balance capacity, collectively exerting critical implications for optimizing long-term outcomes.

#### 2.3.2 Nutritional intervention

Malnutrition constitutes a core pathophysiological mechanism of frailty, with prompt dietary management demonstrating efficacy in mitigating adverse outcome risks. Clinical evidence indicates that 30–80% of hospitalized stroke patients develop dysphagia-associated frailty and malnutrition (19), expert consensus highlights nutrition as a critical strategy for frailty prevention (4). Current nutritional interventions include nutrient supplementation, dietary patterns, and nutritional counseling.

Nutrient supplementation strategies encompass protein and vitamins D, EPA, and DHA. Among these, protein emerges as the sole evidence-backed intervention demonstrating potential as a cornerstone for frailty prevention and management (20). However, meta-analyses of randomized controlled trials (RCTs) reveal that isolated protein supplementation fails to elicit clinically meaningful improvements in muscle mass, strength, or physical performance among frail older adults (21). Protein supplementation combined with exercise workouts may produce even more dramatic results for the frailty elderly.

There are currently four internationally recognized dietary patterns: the Mediterranean dietary pattern, the Japanese dietary pattern, the Western dietary pattern, and the Eastern dietary pattern. The Mediterranean diet is a plant-based diet with olive oil as the main fat, which has been promoted worldwide as the healthiest dietary pattern in recent decades (22). Studies have shown that sustained adherence to this regimen enhances muscular strength, improves somatic functioning, and attenuates risks of geriatric frailty syndrome (23). Guidelines (24) suggest that the traditional Mediterranean diet combined with physical activity is more favorable for the physical recovery of frailty patients. Furthermore, recent research (25) showed that the treatment of ischemic stroke patients with individualized lipid regulation combined with the Mediterranean dietary pattern not only improves the rate of lipid compliance and shortens the time to compliance, but also reduces the rate of stroke recurrence and the incidence of adverse effects, and improves the patient’s prognosis. Another evidence has identified a correlation between frailty and nutritional status in elderly stroke patients (26). This finding suggests that although standardized dietary intervention protocols specifically targeting stroke management have not been established internationally, nutritional interventions implementing Mediterranean dietary regimens may offer dual therapeutic benefits for stroke patients with comorbid frailty - potentially ameliorating both frailty progression and prognostic outcomes. However, the precise clinical efficacy and magnitude of these effects require validation through rigorous prospective studies with standardized outcome metrics.

#### 2.3.3 Cognitive intervention

Cognitive frailty is defined as a heterogeneous clinical syndrome characterized by the concomitant presence of physical frailty and cognitive impairment, excluding Alzheimer’s disease or other dementias (27). And has been established as a significant predictor of both short-and long-term mortality, dementia, disability, and other adverse health outcomes (28). Therefore, when a stroke patient presents with cognitive frailty it also means that the stroke patient with frailty is cognitively impaired.

Cognitive interventions include exercise, nutrition, and cognitive training interventions (29). Current evidence indicates these interventions are predominantly applied through combined protocols in frail older adults, with limited studies isolating their individual therapeutic effects. Recent international research demonstrates that exercise-based interventions constitute the most prevalent non-pharmacological management for cognitive frailty (30). Furthermore, synergistic intervention combining cognitive training with physical exercise has shown superior efficacy in mitigating cognitive frailty compared to unimodal approaches (cognitive training or exercise alone) (31).

Emerging evidence demonstrates that combined resistance band training and cognitive exercise interventions effectively ameliorate physical frailty and enhance cognitive performance in older adults, potentially delaying or reversing the progression of cognitive frailty (32). Observational studies by HUANG Yanan (33) have confirmed a significant correlation between frailty and cognitive impairment, while WANG Ying’s (34) investigation revealed a robust negative correlation between Edmonton Frail Scale (ESF) total scores and Montreal Cognitive Assessment (MoCA) total scores (*r* = −0.491, *p* < 0.001), and the two interact with each other in a constant vicious circle. These findings underscore the clinical imperative to:

1. Systematically monitor cognitive function in stroke survivors using standardized assessments (e.g., MoCA).
2. Implement early cognitive rehabilitation to disrupt the frailty-cognition interaction.
3. Optimize multidimensional interventions for improved long-term outcomes.

#### 2.3.4 Psychosocial intervention

Gobbens’ integrative frailty model conceptualizes frailty as a multidimensional construct encompassing somatic, psychological, and social domains (35). Supporting this framework, research in elderly stroke populations (36) identifies psychological frailty as the second most prevalent manifestation after physical impairment, with its severity demonstrating significant associations with anxiety intensity. Clinically, this psychological frailty syndrome primarily presents through three core components: anxiety disorders, depressive symptoms, and reduced psychological resilience. This multidimensional impairment significantly compromises older adults’ cognitive function, emotional regulation, and coping capacities, adversely affecting disease prognosis and health outcomes. Among the studies on psychological factors of frailty, depression has received the most attention and consistent conclusions (37). Multiple studies have demonstrated a positive correlation between frailty and depression. Scholar Soysal (38) further indicated that interventions targeting either syndrome may effectively prevent the onset of the other.

Targeted psychological interventions can alleviate psychological distress and foster positive coping strategies in patients, thereby mitigating frailty progression and enhancing quality of life among stroke survivors. However, current research on psychological interventions targeting frailty remains limited, with insufficient conceptual clarity regarding psychological frailty itself. Presently, the identification and severity assessment of psychological frailty in older adults predominantly rely on standardized scales. Consequently, there is currently insufficient evidence to conclusively demonstrate that psychological interventions for frail patients directly improve stroke prognosis.

#### 2.3.5 Social intervention

In 2017, Bunt et al. (39) conceptualized social frailty through the lens of the social production function theory, defining it as a persistent state characterized by the absence of one or more critical resources essential for meeting basic social needs, along with deficiencies in social behaviors, social engagement, and self-management capabilities. Compared to physical frailty, less attention has been paid to the perception of social frailty both domestically and internationally, and studies (40) suggest that social frailty in older adults may precede and contribute to physical frailty, and is associated with multiple adverse health outcomes.

#### 2.3.6 Pharmacologic intervention

##### 2.3.6.1 Medication management and risk of frailty

There was a significant positive correlation between polypharmacy and frailty in the elderly (41). Daimaru et al. (42), based on the Itabashi Longitudinal Study of Aging cohort (*n* = 1,021), found that the rate of polypharmacy (≥5 medications/day) in community-dwelling older adults amounted to 30.2%, and that the prevalence of frailty in the polypharmacy group (10.1%) was significantly higher than that of the non-multiple-medication group (5.0%). Studies have shown that polypharmacy is an independent risk factor for frailty phenotypes (e.g., weight loss, decreased muscle strength, and slowed gait speed). In this regard, Thiruchelvam et al. (43) proposed the establishment of a system of regular medication review by pharmacists in collaboration with general practitioners. ICFSR, on the other hand, explicitly recommended (44) that medication management should be implemented in frail patients using the Screening Tool for Older People’s Prescribing (STOPP/START) or the Beers criteria, and that they should be included in a comprehensive multidisciplinary intervention program.

##### 2.3.6.2 Advances in research on targeted pharmacotherapy for frailty

Current pharmacologic interventions for frailty are still in the exploratory phase, and there is no evidence-based consensus on their effectiveness and risk–benefit ratio (45). Although basic research suggests that anti-inflammatory agents (e.g., IL-6 inhibitors) along with hormone modulators (e.g., testosterone) may slow the progression of frailty by improving metabolic homeostasis (46), much of the available clinical evidence stems from observational studies. As noted in a systematic evaluation in 2021 (47), of the 25 pharmacological intervention studies included, 10 individual pharmacological interventions showed improvements in physical fitness, muscle strength, or body composition with the use of alfacalcitol, testosterone, etc., but there was no consistent evidence to support a long-term benefit on the core phenotype of frailty, i.e., there was a lack of prospective, randomized, controlled trials validating a causal association. So far, data on a causal relationship between drugs and frailty are inconclusive or related to single-drug interventions on partial aspects of frailty. Although several drug candidates are currently in phase II clinical trials, no drug has yet been approved by regulatory agencies globally for frail indications (46). Although there are several drug candidates entering phase II clinical trials, no drug has yet been approved by regulatory agencies globally for frail indications (46).

## 3 Discussion

In summary, the most paramount factor is exercise intervention. Studies suggest that physical exercise, particularly group-based exercise programs involving multiple participants, serves as a critical component in addressing social frailty. These interventions not only improve social frailty but also target psychological aspects by alleviating depression and loneliness, enhancing well-being (48, 49), and increasing life satisfaction, thereby mitigating and delaying the progression of frailty. The second key factor is social support. Social support can be further subdivided into three dimensions based on its sources: objective support (tangible assistance), subjective support (perceived emotional support), and support utilization (ability to mobilize resources). Studies have demonstrated that robust social support serves as a protective factor against frailty in community-dwelling older adults (50), helping to preserve autonomy in frail individuals (51) and mitigate frailty severity.

However, due to multifaceted constraints (e.g., resource allocation disparities and cultural barriers), the current status of social support for frail older adults in China remains suboptimal. In this context, Tu et al. (52) proposed a series of measures to improve social support for frail elderly individuals. Through social interventions targeting both subjective and objective social support, the initiatives aimed to enhance the level and utilization of social support among frail older adults, thereby delaying or even reversing the progression of frailty and reducing the incidence of adverse health outcomes. The suggested intervention modalities and frequency for the different intervention types, and the key benefits generated can be seen in Table 1.

**Table 1:** Summary of the 6 intervention types.

Intervention type	Key components	Recommended frequency/duration	Primary benefits	References
Exercise	Resistance training + Aerobic exercise + Balance training (e.g., Otago Exercise Program)	2–3 sessions/week, 50–60 min/session, 8 weeks duration	Significantly improves muscle strength, gait speed, and reduces frailty severity	(8–11, 17)
Nutrition	Mediterranean diet + Protein supplementation (1.2 g/kg/day)	≥6 months duration	Enhances nutritional status and mitigates frailty progression	(20–26)
Cognitive	Resistance band training + Cognitive tasks (e.g., memory training)	3 sessions/week, 30 min/session, 12 weeks duration	Improves cognitive function and promotes reversal of frailty phenotypes	(30–34)
Psychosocial	Group cognitive-behavioral therapy + Social activities	Weekly sessions, 3 months duration	Reduces depressive symptoms and enhances social support utilization	(36–40)
Pharmacologic	Medication review (STOPP/ START criteria) + Testosterone supplementation	Quarterly medication review	Reduces polypharmacy rates and frailty-related adverse events	(42–47)
Multimodal	Combined exercise, nutrition, and cognitive protocols	Twice weekly multidisciplinary sessions, 6 months duration	Improves activities of daily living (ADL) and reduces rehospitalization rates	(17, 49)

Although current research on stroke and social frailty remains limited, and interventions targeting social frailty are relatively scarce, existing studies have confirmed that social frailty serves as an independent risk factor for cognitive impairment in older adults (53). Interventions addressing social frailty in the elderly population could potentially prevent the progression of physical frailty and cognitive decline (54), thereby playing a crucial role in maintaining and improving quality of life. While direct evidence demonstrating the positive impact of improved social frailty on health outcomes in stroke patients is currently unavailable, indirect evidence from these studies suggests that social interventions targeting frail elderly individuals may yield beneficial effects. These findings indirectly suggest that enhancing social support utilization and optimizing multidimensional social support systems may, to some extent, improve prognostic outcomes in stroke patients.

## 4 Conclusion

An Italian study (7) revealed that stroke is the only cerebrovascular disease associated with frailty syndrome, with the prevalence of frailty in stroke patients being twice that of non-stroke individuals. Soysal (38) posited that interventions targeting one of the two syndromes may prevent the onset of the other. This suggests that proactive frailty interventions could improve prognostic outcomes in stroke patients. Current evidence emphasizes non-pharmacological interventions for frailty management (55), including exercise, nutritional support, cognitive training, and psychosocial interventions, with exercise being the most widely recommended approach in clinical guidelines. A multimodal intervention combining exercise with nutritional, cognitive, and psychosocial strategies should be prioritized for stroke patients to optimize intervention efficacy, mitigate adverse health outcomes of frailty, and ultimately enhance quality of life and longevity. However, existing studies on frailty interventions in stroke patients remain limited, and insufficient evidence exists to quantify the strength of frailty’s impact on stroke progression. Further validation through rigorous research is warranted.

### 4.1 Limitation and future directions

This study has three primary limitations: Substantial heterogeneity across studies: Significant variability in study designs (e.g., randomized controlled trials [RCTs] versus observational studies), sample sizes (ranging from 100 to 1,021 participants), and outcome measures (e.g., MoCA scores versus frailty indices) introduced potential bias during pooled analyses, compromising the reliability of conclusions.

Unverified applicability of interventions: The cost-effectiveness and cultural adaptability of part interventions (e.g., the Otago Exercise Program and traditional Chinese exercises) remain inadequately validated, necessitating further empirical support for their broader implementation.

Insufficient pharmacological evidence: Current evidence lacks high-quality studies elucidating the impact of pharmacological interventions on post-stroke frailty. Specifically, there is a paucity of prospective randomized controlled trials targeting core pathological mechanisms of stroke-related frailty, hindering definitive assessments of risk–benefit ratios. Furthermore, future multicenter phase III clinical trials are imperative to validate targeted therapeutic strategies through dynamic biomarker monitoring.

Future research should focus on: Developing dynamically adjusted exercise intensity-frequency algorithms by integrating biomarkers and wearable device data to optimize long-term adherence; Conducting feasible multicenter, double-blind RCTs enrolling stroke patients with well-defined frailty phenotypes, using composite endpoints (e.g., frailty index reduction + ADL improvement) to evaluate long-term drug safety and efficacy; Assessing cost-effectiveness of simplified Otago Exercise Program versions (e.g., home-based self-training with remote guidance) in resource-limited settings; Designing cross-cultural adaptation trials (e.g., multinational RCTs in Western cohorts) for traditional Chinese exercises (Tai Chi, Baduanjin) to validate efficacy variations and optimize implementation strategies.

## Footnotes

^1^The Borg CR-10 scale is a validated method to quantify the effects of different types of exercise training, and Brandt et al. categorized the results of the Borg scale as mild (Borg ≤ 2), moderate (Borg > 2 - < 5), severe (Borg ≥ 5 - < 7), and near maximal (Borg ≥ 7).

## References

[1] Chang L He X Cao X Zhang Z Peng X Mei B. Nao Zhong fang Zhi Ke Pu Xuan Jiao Zhuan Jia Gong Shi [expert consensus on popular science education for stroke prevention and treatment]. Zhong Feng Yu Shen Jing Ji Bing. (2021) 28:713–8.

[2] Wang L Peng B Zhang H Wang Y Liu M Shan C. Brief report on stroke prevention and treatment in China, 2020. Chin J Cerebrovasc Dis. (2022) 19:136–44.

[3] Huang Y Wang X Chen Y Su W Chen X Yan F. Nao Zhong Huan Zhe Bing he Shuai Ruo de Yan Jin Jin Zhan [research Progress on frailty in stroke patients]. Zhong Guo Hu Li Guan Li. (2023) 23:282–6.

[4] Chinese Geriatrics Society, Editorial Board of Zhong Hua Lao Nian Yi Xue Za Zhi. Lao Nian Ren Shuai Ruo Yu fang Zhong Guo Zhuan Jia Gong Shi (2022) [Chinese expert consensus on frailty prevention in older adults (2022)]. Zhonghua Lao Nian Yi Xue Za Zhi. (2022) 41:503–11. doi: 10.3760/cma.j.issn.0254-9026.2022.05.001

[5] Li A. Que Xue Xing Nao Zhong Huan Zhe Shuai Ruo de Ying Xiang yin Su Ji Gan Yu Cuo Shi de Yan Jiu Jin Zhan [influencing factors and intervention measures of frailty in ischemic stroke patients: a research Progress]. Xun Zheng Hu Li. (2023) 9:811–4. doi: 10.12102/j.issn.2095-8668.2023.05.009

[6] Joyce N Atkinson T Mc Guire K Wiggam MI Gordon PL Kerr EL. Frailty and stroke thrombectomy outcomes—an observational cohort study. Age Ageing. (2022) 51:afab260. doi: 10.1093/ageing/afab260, PMID: 35150584

[7] Palmer K Vetrano DL Padua L Romano V Rivoiro C Scelfo B. Frailty syndromes in persons with cerebrovascular disease: a systematic review and meta-analysis. Front Neurol. (2019) 10:1255. doi: 10.3389/fneur.2019.01255, PMID: 31849819 PMC6896936

[8] Bray NW Smart RR Jakobi JM Jones GR. Exercise prescription to reverse frailty. Appl Physiol Nutr Metab. (2016) 41:1112–6. doi: 10.1139/apnm-2016-0226, PMID: 27649859

[9] Zhao W Zhang B Sun T Zou H Lv Y Liu Y. Yun Dong Gai Shan Lao Nian Ren Shuai Ruo Zhuang Kuang de Ji Liang Xiao Ying Meta fen xi [dose-response effect of exercise on frailty in older adults: a Meta-analysis]. Zhong Guo Man Xing Bing Yu Fang Yu Kong Zhi. (2023) 31:860–6. doi: 10.16386/j.cjpccd.issn.1004-6194.2023.11.013

[10] Saragih ID Yang YP Saragih IS Batubara SO Lin CJ. Effects of resistance bands exercise for frail older adults: a systematic review and meta-analysis of randomised controlled studies. J Clin Nurs. (2022) 31:43–61. doi: 10.1111/jocn.15950, PMID: 34289511

[11] Talar K Hernández-Belmonte A Vetrovsky T Steffl M Kałamacka E Courel-Ibáñez J. Benefits of resistance training in early and late stages of frailty and sarcopenia: a systematic review and meta-analysis of randomized controlled studies. J Clin Med. (2021) 10:1630. doi: 10.3390/jcm10081630, PMID: 33921356 PMC8070531

[12] Penna LG Pinheiro JP Ramalho SHR Ribeiro CF. Effects of aerobic physical exercise on neuroplasticity after stroke: systematic review. Arq Neuropsiquiatr. (2021) 79:832–43. doi: 10.1590/0004-282X-ANP-2020-0551 34669820

[13] Li C Li X. Ta Che you Xun Xun Lian Pei he Chang Guan Liao fa dui Lao Nian Ji Xing que Xue Xing Nao Zhong Huan Zhe Yu Hou de Ying Xiang [effects of cycling aerobic training combined with conventional therapy on prognosis of elderly patients with acute ischemic stroke]. Zhonghua Lao Nian Xin Nao Xue Guan Bing Za Zhi. (2023) 25:648–50. doi: 10.3969/j.issn.1009-0126.2023.06.022

[14] Ge L Zheng QX Liao YT Tan JY Xie QL Rask M. Effects of traditional Chinese exercises on the rehabilitation of limb function among stroke patients: a systematic review and meta-analysis. Complement Ther Clin Pract. (2017) 29:35–47. doi: 10.1016/j.ctcp.2017.08.005, PMID: 29122267

[15] Campbell AJ Robertson MC Gardner MM Norton RN Tilyard MW Buchner DM. Randomised controlled trial of a general practice programme of home based exercise to prevent falls in elderly women. BMJ. (1997) 315:1065–9. doi: 10.1136/bmj.315.7115.1065, PMID: 9366737 PMC2127698

[16] Pei Z Wang C Meng X. Ao ta Ge Yun Dong Xiang mu dui Nao Zhong Huan Zhe die Dao Gan Yu Xiao Guo de Meta fen xi [intervention effect of Otago exercise program on falls in stroke patients: a Meta-analysis]. Kang Fu Xue Bao. (2019) 29:60–6. doi: 10.3724/SP.J.1329.2019.02060

[17] Yang X Zhao C Li Y Liu X Wang K Xu L. Nao Zhong Huan Zhe Yun Dong Kang Fu de Zui Jia Zheng Ju Zong Jie [best evidence summary for exercise rehabilitation in stroke patients]. Zhong Guo Shi Yong Hu Li Za Zhi. (2023) 39:915–23. doi: 10.3760/cma.j.cn211501-20220914-02866

[18] Chu J Chen X Yan g. The interpretation of the Asia-Pacific clinical practice guidelines for the management of frailty. Chin J Geriatr. (2019) 38:1213–5. doi: 10.3760/cma.j.issn.0254-9026.2019.11.005

[19] Pontes ÉS Amaral AK Rêgo FL Azevedo EH Silva PO. Quality of life in swallowing of the elderly patients affected by stroke. Arq Gastroenterol. (2017) 54:27–32. doi: 10.1590/s0004-2803.2017v54n1-05, PMID: 28079235

[20] Wu J Wang C Wei Z Cui M. Ying Yang Gan Yu Yu Lao Nian Shuai Ruo Xiang Guan Xing de Yan Jiu Jin Zhan [research Progress on the relationship between nutritional intervention and frailty in older adults]. Guo Ji Lao Nian Yi Xue Za Zhi [Int J Geriatr]. (2022) 43:483–6. doi: 10.3969/j.issn.1674-7593.2022.04.024

[21] Oktaviana J Zanker J Vogrin S Duque G. The effect of protein supplements on functional frailty in older persons: a systematic review and meta-analysis. Arch Gerontol Geriatr. (2020) 86:103938. doi: 10.1016/j.archger.2019.103938, PMID: 31726309

[22] Mazza E Ferro Y Pujia R Mare R Maurotti S Montalcini T. Mediterranean diet in healthy aging. J Nutr Health Aging. (2021) 25:1076–83. doi: 10.1007/s12603-021-1675-6, PMID: 34725664 PMC8442641

[23] Guan J Zhao M Fang Y. Di Zhong Hai yin Shi Yu Lao Nian Shuai Ruo Zong he Zheng Xiang Guan Xing de Meta fen xi [correlation between Mediterranean diet and frailty syndrome in older adults: a Meta-analysis]. Shi Yong Lao Nian Yi Xue [Practical Geriatr]. (2020) 34:1127–31. doi: 10.3969/j.issn.1003-9198.2020.11.007

[24] Zhang Y Niu Y Wang D Zheng Y. 2019 Nian ICFSR Guo Ji Lin Chuang Shi Jian Zhi nan Jie Du Ji dui wo Guo Lao Nian Ren Shuai Ruo Shi Bie Ji Guan Li de qi Shi [interpretation of the 2019 ICFSR international clinical practice guidelines and implications for frailty identification and Management in Chinese Older Adults]. Hu Li Yan Jiu [Nursing Res]. (2020) 34:2433–6. doi: 10.12102/j.issn.1009-6493.2020.14.001

[25] Bao Z Wu Y Zhang L Qian X. Xue Zhi Ge Ti Hua Tiao Kong Lian he Di Zhong Hai yin Shi Mo Shi Zhi Liao que Xue Xing Nao Zhong 100 Li [individualized lipid control combined with Mediterranean diet for ischemic stroke: a study of 100 cases]. Anhui Yi Yao. (2022) 26:1640–4. doi: 10.3969/j.issn.1009-6469.2022.08.036

[26] Lin L Yeh H Wu J Feng X. Lao Nian Nao Zhong Huan Zhe Shuai Ruo Yu Ying Yang Feng Xian de Xiang Guan Xing Yan Jiu [correlation between frailty and nutritional risk in elderly stroke patients]. Guo Ji Yi Yao Wei Sheng Dao Bao. (2021) 27:3243–7. doi: 10.3760/cma.j.issn.1007-1245.2021.20.034

[27] Arai H Satake S Kozaki K. Cognitive frailty in geriatrics. Clin Geriatr Med. (2018) 34:667–75. doi: 10.1016/j.cger.2018.06.011, PMID: 30336994

[28] KelaiditiECesariMCanevelliMvan Abellan KanGOussetP-JGillette-GuyonnetS. Cognitive frailty: rational and definition from an (IANA/IAGG) international consensus group. J Nutr Health Aging. (2013) 17:726–34. doi: 10.1007/s12603-013-0367-224154642

[29] Gong W Zhang Y. Ren Zhi Shuai Ruo Kang Fu Zhong Guo Zhuan Jia Gong Shi 2023 [Chinese expert consensus on cognitive frailty rehabilitation (2023)]. Zhong Guo Yi Kan. (2023) 58:949–53. doi: 10.3969/j.issn.1008-1070.2023.09.007

[30] Li X Zhang Y Tian Y Cheng Q Gao Y Gao M. Exercise interventions for older people with cognitive frailty—a scoping review. BMC Geriatr. (2022) 22:721. doi: 10.1186/s12877-022-03370-3, PMID: 36045320 PMC9434944

[31] Kwan RYC Lee D Lee PH Tse M Cheung DSK Thiamwong L. Effects of an mHealth brisk walking intervention on increasing physical activity in older people with cognitive frailty: pilot randomized controlled trial. JMIR Mhealth Uhealth. (2020) 8:e16596. doi: 10.2196/16596, PMID: 32735218 PMC7428907

[32] Han J Wang J Gao J Xie B. Tan Li Dai Yun Dong Lian he Ren Zhi Xun Lian dui she Qu Lao Nian Ren Ren Zhi Shuai Ruo de Gan Yu Xiao Guo [effect of elastic band exercise combined with cognitive training on cognitive frailty in community-dwelling older adults]. Hu Li Yan Jiu. (2022) 36:624–9. doi: 10.12102/j.issn.1009-6493.2022.04.010

[33] Huang Y. Lao Nian Nao Zhong Huan Zhe Shuai Ruo Xian Zhuang Ji Qi Yu Zhong Hou Ren Zhi Zhang Ai de Xiang Guan Xing Yan Jiu [Frailty Status and Its Correlation with Post-Stroke Cognitive Impairment in Elderly Stroke Patients]. Lanzhou: Lanzhou University (2023).

[34] Wang Y Tao X Dong K Song H Hu W. Ji Xing que Xue Xing Nao Zhong Huan Zhe Shuai Ruo Yu Ren Zhi Gong Neng Zhang Ai de Xiang Guan Xing [correlation between frailty and cognitive dysfunction in acute ischemic stroke patients]. Zhong Guo Yi Yao Dao Bao. (2021) 18:82–4.

[35] Gobbens RJ Luijkx KG Wijnen-Sponselee MT Schols JM. In search of an integral conceptual definition of frailty: opinions of experts. J Am Med Dir Assoc. (2010) 11:338–43. doi: 10.1016/j.jamda.2009.09.015, PMID: 20511101

[36] Liu L. Lao Nian Nao Zhong Huan Zhe Shuai Ruo Xian Zhuang Ji qi Ying Xiang yin Su Yan Jiu [frailty status and influencing factors in elderly stroke patients]. Changsha: Hunan University of Chinese Medicine (2022).

[37] Shi J Shen G Yu P. Lao Nian Ren Ren Zhi he she hui Xin Li Shuai Ruo [cognitive and psychosocial frailty in older adults]. Zhong Guo Lin Chuang Bao Jian Za Zhi. (2022) 25:762–6. doi: 10.3969/J.issn.1672-6790.2022.06.010

[38] Soysal P Veronese N Thompson T Kahl KG Fernandes BS Prina AM. Relationship between depression and frailty in older adults: a systematic review and meta-analysis. Ageing Res Rev. (2017) 36:78–87. doi: 10.1016/j.arr.2017.03.005, PMID: 28366616

[39] Bunt S Steverink N Olthof J van der Schans CP Hobbelen JSM. Social frailty in older adults: a scoping review. Eur J Ageing. (2017) 14:323–34. doi: 10.1007/s10433-017-0414-7, PMID: 28936141 PMC5587459

[40] Makizako H Shimada H Doi T Tsutsumimoto K Hotta R Nakakubo S. Social frailty leads to the development of physical frailty among physically non-frail adults: a four-year follow-up longitudinal cohort study. Int J Environ Res Public Health. (2018) 15:490. doi: 10.3390/ijerph15030490, PMID: 29534470 PMC5877035

[41] Derhem B Özsari S. Frailty and polypharmacy in primary care. Biol Res Nurs. (2023) 25:658–63. doi: 10.1177/10998004231179485, PMID: 37231714

[42] Daimaru K Osuka Y Kojima N Mizukami K Motokawa K Iwasaki M. Associations of polypharmacy with frailty severity and each frailty phenotype in community-dwelling older adults: Itabashi longitudinal study on aging. Geriatr Gerontol Int. (2024) 24:196–201. doi: 10.1111/ggi.14789, PMID: 38169078

[43] Thiruchelvam K Byles J Hasan SS Egan N Kairuz T. Prevalence and association of continuous polypharmacy and frailty among older women: a longitudinal analysis over 15 years. Maturitas. (2021) 146:18–25. doi: 10.1016/j.maturitas.2021.01.005, PMID: 33722360

[44] Dent E Morley JE Cruz-Jentoft AJ Woodhouse L Rodríguez-Mañas L Fried LP. Physical frailty: ICFSR international clinical practice guidelines for identification and management. J Nutr Health Aging. (2019) 23:771–87. doi: 10.1007/s12603-019-1273-z, PMID: 31641726 PMC6800406

[45] Pahor M Kritchevsky SB Waters DL Villareal DT Morley J Hare JM. Designing drug trials for frailty: ICFSR task force 2018. J Frailty Aging. (2018) 7:150–4. doi: 10.14283/jfa.2018.20, PMID: 30095144

[46] Cesari M Bernabei R Vellas B Fielding RA Rooks D Azzolino D. Challenges in the development of drugs for sarcopenia and frailty-report from the international conference on frailty and sarcopenia research (ICFSR) task force. J Frailty Aging. (2022) 11:135–42. doi: 10.14283/jfa.2022.30, PMID: 35441189 PMC9017069

[47] Pazan F Petrovic M Cherubini A onder G Cruz-Jentoft AJ Denkinger M. Current evidence on the impact of medication optimization or pharmacological interventions on frailty or aspects of frailty: a systematic review of randomized controlled trials. Eur J Clin Pharmacol. (2021) 77:1–12. doi: 10.1007/s00228-020-02951-8, PMID: 32770278 PMC8197722

[48] Makizako H Tsutsumimoto K Shimada H Arai H. Social frailty among community-dwelling older adults: recommended assessments and implications. Ann Geriatr Med Res. (2018) 22:3–8. doi: 10.4235/agmr.2018.22.1.3, PMID: 32743237 PMC7387641

[49] Tarazona-Santabalbina FJ Gómez-Cabrera MC Pérez-Ros P Martínez-Arnau FM Cabo H Tsaparas K. A multicomponent exercise intervention that reverses frailty and improves cognition, emotion, and social networking in the community-dwelling frail elderly: a randomized clinical trial. J Am Med Dir Assoc. (2016) 17:426–33. doi: 10.1016/j.jamda.2016.01.019, PMID: 26947059

[50] Lei P Liu C Gao Y Xue M. Xin Li she hui yin Su Yu she Qu Lao Nian Ren Shuai Ruo de Xiang Guan Xing Yan Jiu [association between psychosocial factors and frailty in community-dwelling older adults]. Zhong Guo Quan Ke Yi Xue. (2018) 21:180–5. doi: 10.3969/j.issn.1007-9572.2018.02.12

[51] Dent E Martin FC Bergman H Woo J Romero-Ortuno R Walston JD. Management of frailty: opportunities, challenges, and future directions. Lancet. (2019) 394:1376–86. doi: 10.1016/S0140-6736(19)31785-4, PMID: 31609229

[52] Tu H Zhang S Fang Y Shen J Wan X He G. Shuai Ruo Lao Nian Ren she hui Zhi chi Yan Jiu Jin Zhan [research Progress on social support in frail older adults]. Hu Li Yan Jiu. (2023) 37:1988–91. doi: 10.12102/j.issn.1009-6493.2023.11.019

[53] Tsutsumimoto K Doi T Makizako H Hotta R Nakakubo S Makino K. Association of social frailty with both cognitive and physical deficits among older people. J Am Med Dir Assoc. (2017) 18:603–7. doi: 10.1016/j.jamda.2017.02.004, PMID: 28411094

[54] Fan J Liu Y Zhao H Kong L Mao J Li J. Lao Nian Ren she hui Shuai Ruo de Yan Jiu Jin Zhan [research Progress on social frailty in older adults]. Hu Li Xue Za Zhi. (2020) 35:106–9. doi: 10.3870/j.issn.1001-4152.2020.02.106

[55] Zhang J Zhang D Wang H. Shuai Ruo Lao Nian Ren Fei Yao Wu Gan Yu de Yan Jiu Jin Zhan [research Progress on non-pharmacological interventions for frail older adults]. Zhonghua Hu Li Za Zhi. (2020) 55:1588–93. doi: 10.3761/j.issn.0254-1769.2020.10.026

