# Correction: A narrative review of interventions for post-stroke frailty: current advances and future directions

**DOI:** 10.3389/fneur.2025.1702913

**Published:** 2025-09-22

**Authors:** Xiaowen Fan, Yi Xia, Shengwang Xu, Shulei Jia

**Affiliations:** ^1^School of Nursing, Nanchang University, Nanchang, Jiangxi, China; ^2^Department of Breast Surgery, The Second Affiliated Hospital of Nanchang University, Nanchang, Jiangxi, China

**Keywords:** frail, stroke, intervention, review, exercise, nutrition

A correction on the author's affiliations. Affiliation 1 will be replaced by the original affiliation 2 and affiliation 2 will be replaced by the original affiliation 1. Authors Xiaowen Fan, Yi Xia and Shulei Jia are affiliated to “School of Nursing, Nanchang University, Nanchang, Jiangxi, China” and author Shengwang Xu are affiliated to “Department of Breast Surgery, The Second Affiliated Hospital of Nanchang University, Nanchang, Jiangxi, China”.

The original version of this article has been updated.

