# Correction: A narrative review of interventions for post-stroke frailty: current advances and future directions

**DOI:** 10.3389/fneur.2025.1702288

**Published:** 2025-10-03

**Authors:** Xiaowen Fan, Yi Xia, Shengwang Xu, Shulei Jia

**Affiliations:** ^1^School of Nursing, Nanchang University, Jiangxi Medical College, Nanchang, Jiangxi, China; ^2^Department of Breast Surgery, The Second Affiliated Hospital of Nanchang University, Nanchang, Jiangxi, China

**Keywords:** frail, stroke, intervention, review, exercise, nutrition

Xiaowen Fan's affiliation [School of Nursing, Nanchang University, Jiangxi Medical College, Jiangxi Province] was erroneously given as [School of Nursing, Nanchang University, Nanchang, Jiangxi, China].

The original version of this article has been updated.

